# ALV-J infection induces chicken monocyte death accompanied with the production of IL-1β and IL-18

**DOI:** 10.18632/oncotarget.21906

**Published:** 2017-10-13

**Authors:** Manman Dai, Min Feng, Tingting Xie, Yuanfang Li, Zhuohao Ruan, Meiqing Shi, Ming Liao, Xiquan Zhang

**Affiliations:** ^1^ College of Veterinary Medicine, South China Agricultural University, Guangzhou, Guangdong, China; ^2^ Department of Animal Genetics, Breeding and Reproduction, College of Animal Science, South China Agricultural University, Guangzhou, Guangdong, China; ^3^ Guangdong Provincial Key Lab of Agro-Animal Genomics and Molecular Breeding, Key Lab of Chicken Genetics, Breeding and Reproduction, Ministry of Agriculture, Guangzhou, Guangdong, China; ^4^ Division of Immunology, Virginia-Maryland Regional College of Veterinary Medicine, University of Maryland, College Park, Maryland, United States of America

**Keywords:** ALV-J, monocyte, pyroptosis, immunosuppression, chicken macrophage

## Abstract

Immunosuppression induced by avian leukosis virus subgroup J (ALV-J) causes serious reproduction problems and secondary infections in chickens. Given that monocytes are important precursors of immune cells including macrophages and dendritic cells, we investigated the fate of chicken monocytes after ALV-J infection. Our results indicated that most monocytes infected with ALV-J including field or laboratory strains could not successfully differentiate into macrophages due to cells death. And cells death was dependent upon viral titer and accompanied with increased IL-1β and IL-18 mRNA levels. In addition, ALV-J infection up-regulated caspase-1 and caspase-3 activity in monocytes. Collectively, we found that ALV-J could cause cell death in chicken monocytes, especially pyroptosis, which may be a significant reason for ALV-J induced immunosuppression.

## INTRODUCTION

Avian leukosis virus (ALV) is a chicken retrovirus that induces neoplastic disease and immunosuppression, and is an important factor in avian viral coinfections [1–3]. ALV subgroup J (ALV-J) infections greatly enhance the probability of a secondary infection through virally-induced immunosuppression. Our previous studies demonstrated that ALV-J induces host innate immune responses in chicken’s primary monocyte-derived macrophages suggesting that macrophages are important in ALV-J associated immune defense and escape [4]. ALV-J infection also inhibits differentiation and maturation of chicken bone marrow-derived dendritic cells (BM-DC) and triggers their apoptosis [5, 6].

Monocytes are circulating precursors of tissue DCs and macrophages [7]. There have been reports that Dengue virus infection induces pyroptosis in human monocytes [8]. However, little is known about the fate of chicken monocytes after ALV infection. We speculate that infection of primary monocytes with ALV-J in chickens may lead to monocyte death.

Pyroptosis has been long regarded as caspase-1-mediated, pro-inflammatory form of programmed cell death and occurs in both macrophage and non-macrophage cells [9–11]. It is mainly initiated by NOD-like receptors (NLRs). After activation, pyroptosis-related inflammasome, including NLRPlb, NLRP3 and NLRC4 will form and lead to a direct activation of caspase-1 [12]. Caspase-1 is a key player in the pyroptosis pathway, cleaving and releasing pro-inflammatory cytokines IL-1β and IL-18 which contribute to a rapid loss of plasma membrane integrity [13, 14]. Activation of caspase-1 results in cell lysis and releases of cellular contents into the extracellular environment.

Interestingly, caspase-1 is not involved in apoptosis, and caspase-1-deficient mice have no defects in apoptosis [15]. In general, caspase-3, caspase-6 and caspase-8 are regarded as the apoptotic caspases, but are not involved in pyroptosis [16]. However, some new studies find that caspase-3 activation can trigger pyroptosis through cleaving Gasdermin E (GSDME) [17, 18]. The gasdermin family including GSDMA, GSDMB ,GSDMC, GSDMD, GSDME (DFNA5), and DFNB59 becomes a new research field on pyroptosis functions in immunity and disease [19]. In particular, GSDMD has been identified as the pyroptosis executioner. Caspase-1 and Caspase-11/4/5 cleave GSDMD to trigger pyroptosis [10, 20]. Thus, some researchers redefine pyroptosis is gasdermin-mediated programmed necrotic cell death [19].

Previous research has tried to explain the mechanism of immunosuppression induced by ALV-J infection, where ALV-J infection intervenes DCs differentiation and induces DC apoptosis [5]. In this study, we attempted to test the effect of ALV-J infection on induction of chicken primary monocyte death, especially pyroptosis. We hope that our findings contribute to the understanding of the mechanisms underlying the development of ALV-J associated immunosuppression disease.

## RESULTS

### Detection of clinical chickens infected with ALV-J

**Figure 1:**
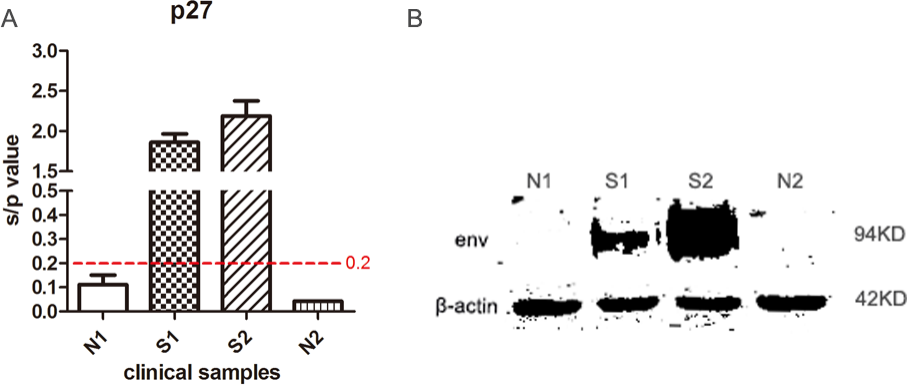
Detection of ALV-J in clinical samples (**A**) ALV-J viremia was detected by measuring the expression levels of ALV group-specific antigen p27 from DF1 cell culture supernatants collected at 7 dpi. (**B**) Expression levels of the ALV-J envelope protein in chicken monocytes were detected by Western blotting using mouse antibody JE9. N1 and N2 represented control healthy chickens; S1 and S2 represented sick chickens infected with ALV.

To confirm whether the Chinese yellow chickens S1 and S2 used in the study were infected with ALV, we monitored viremia for three weeks by incubating plasma samples into DF1 cells. The results indicated that ALV group-specific antigen (p27) of S1 and S2 cultures was positive as detected by ELISA, of which s/*p value* exceeded the threshold value (0.2). On the contrary, the s/*p value* of uninfected chickens (N1 and N2) is below the threshold value (Figure 1A). These results suggested that chickens S1 and S2 were infected with exogenous ALV, but chickens N1 and N2 were not.

Western blot analysis confirmed the expression of ALV-J envelope protein in monocytes isolated from chickens S1 and S2, which was negative in monocytes from chicken N1 and N2 (Figure 1B). Furthermore, we excluded Marek’s disease virus (MDV), reticuloendotheliosis virus (REV) and other subgroup ALV infections by specific PCR [21] and analyzed *env* sequence of ALV strain S1 and S2 (Supplementary Figure 1). Based on the above results, we demonstrated that the ALV strain S1 and S2 are indeed ALV-J, and chicken N1 and N2 are ALV negative. In addition, the virus titres of S1 and S2 were 1.6 × 10^3^ TCID_50_/0.1 mL and 5.0 ×10^3^ TCID_50_/0.1 mL respectively, measured by the method of Reed & Muench [22].

### Observation of clinical chicken monocytes differentiation

**Figure 2:**
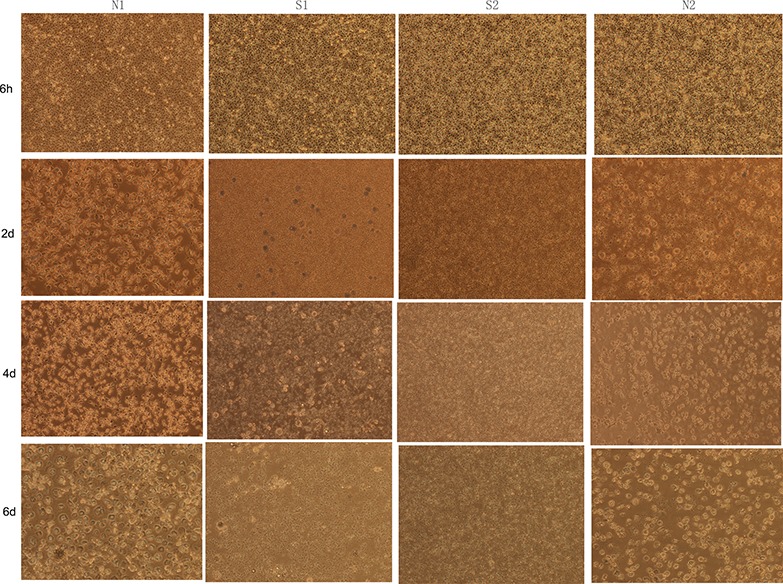
The differentiation state of monocytes isolated from clinical chicken Images of chicken monocytes were taken every 2 d (magnification: 150 ×). N1 and N2 represented uninfected chickens; S1 and S2 represented sick chickens infected with ALV.

We next examined whether the monocytes from the four clinical chickens displayed any differences in their differentiation patterns. In our culture system, adherent monocytes isolated from S1, S2, N1 and N2 showed no obvious difference at 6 h (Figure 2). Monocytes isolated from uninfected chickens N1 and N2 were differentiated at day 2 and showed clear and distinct macrophage-like morphologies at day 6 (Figure 2), in contrast to the infected chicken monocytes that were almost complete lack of differentiation and most of the cells appeared dead (Figure 2). These results suggested that ALV-J infection led to chicken monocytes death.

**Figure 3:**
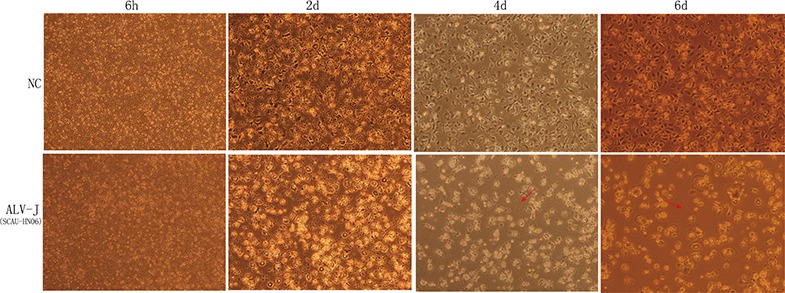
The differentiation state of monocytes isolated from SPF chicken Images taken at 6 h, 2 d, 4 d and 6 d post infection (magnification: 150 ×). NC was the natural control of monocyte without SCAU-HN06 infection. Dead monocyte and cell fragmentation were indicated with the red arrow.

We repeated the preceding experiments by infecting peripheral blood monocytes isolated from SPF chickens infected with ALV-J laboratory strain SCAU-HN06. The uninfected monocytes differentiated into macrophages within the 6-day culture period (Figure 3). However, only a small number of monocytes exhibited some forms of differentiation into macrophages, and there was evidence for extensive cell death in the cultures (Figure 3). These results showed that ALV-J infection resulted in cell death of chicken monocytes.

### Analysis of chicken monocyte death induced by ALV-J

**Figure 4:**
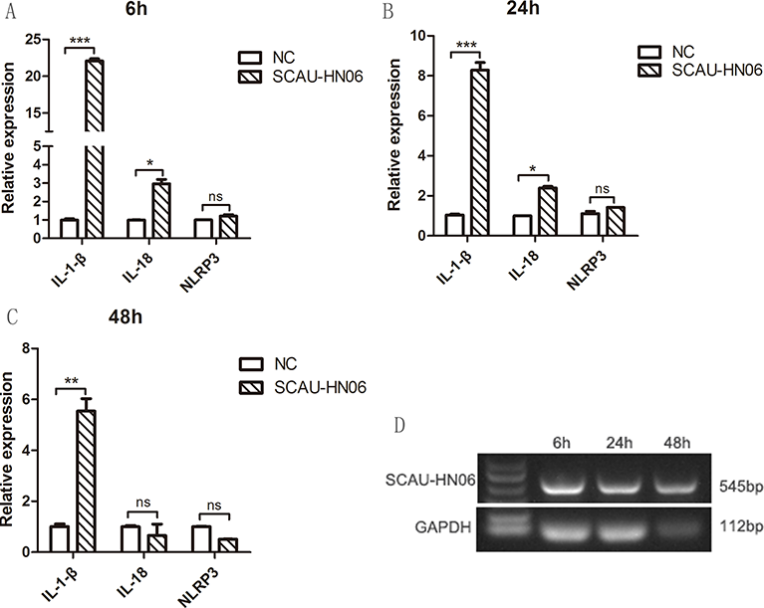
Detection of pyroptosis-related cytokines and sensor expression in monocytes infected with ALV-J strain SCAU-HN06 Monocytes isolated from SPF chicken were infected with SCAU-HN06 (10^4.5^ TCID_50_/0.1 mL). IL-1β, IL-18 and NLRP3 mRNA levels were analyzed using qPCR at (**A**) 6 hpi, (**B**) 24 hpi and (**C**) 48 hpi. (**D**) Infected monocytes at 6, 24 and 48 hpi were collected for RT-PCR using ALV-J specific primers for SCAU-HN06. NC represents the normal monocytes. ^*^P < 0.05; ^**^P < 0.01; ^***^P < 0.001; and ns, not significant.

We further examined the cause of cell death with ALV-J infection via measuring the mRNA expression levels of pyroptosis related cytokine. As shown in Figure 4D, chicken monocytes isolated from SPF chicken were successfully infected by ALV-J strain SCAU-HN06 from 6 h to 48 h. And the transcription levels of IL-1β were significantly increased from 6 to 48 hpi (Figure 4A). Similarly, the level of IL-18 mRNA increased at 6 and 24 hpi (Figure 4B). However, there was no statistical difference in the steady state levels of NLRP3 mRNA (Figure 4C). These findings suggested that monocyte death may be due to pyroptosis.

**Figure 5:**
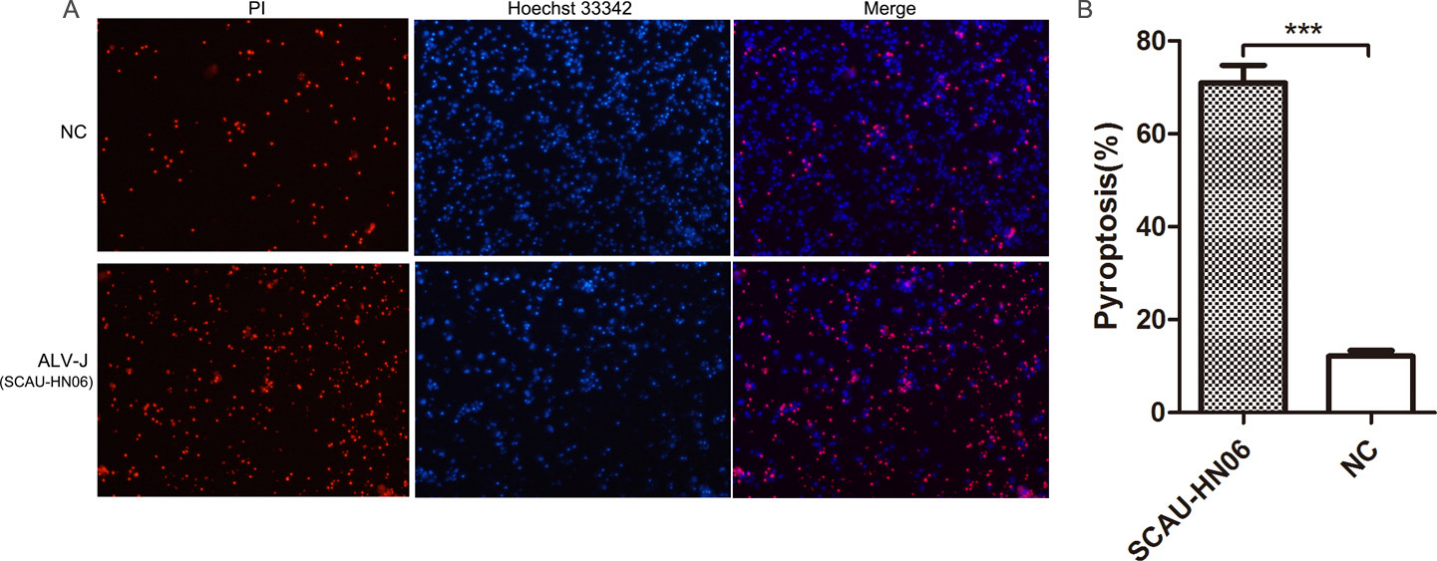
Pyroptosis assay of monocytes infected with ALV-J strain SCAU-HN06 (**A**) Monocytes isolated from SPF chickens were infected with SCAU-HN06 (10^4.5^ TCID_50_/0.1 mL). Uninfected monocytes represented natural control (NC). Cells were stained by propidium iodide (PI) (red indicated pyroptotic cells) and Hoechst 33342 (blue indicated all cells) at 24 hpi. Images were captured and merged using fluorescence microscopy (300 ×). Three independent experiments were performed (each in triplicate), and representative data from one experiment was shown. (**B**) Pyroptosis of five random images containing all cells were calculated. ^***^P < 0.001.

Another assay for cell death was used for confirmation of these results. We determined the ratio of the number of Propidium Iodide (PI) permeable cells (dead monocytes) to total cell numbers as indicated by Hoechst 33342 staining. Uninfected control cells showed less PI staining compared with ALV-J infected cells (Figure 5A). Quantitatively, there was a 71.0% death rate in monocytes at 24 hpi, which was significantly higher than the rate in control monocytes (Figure 5B).

**Figure 6:**
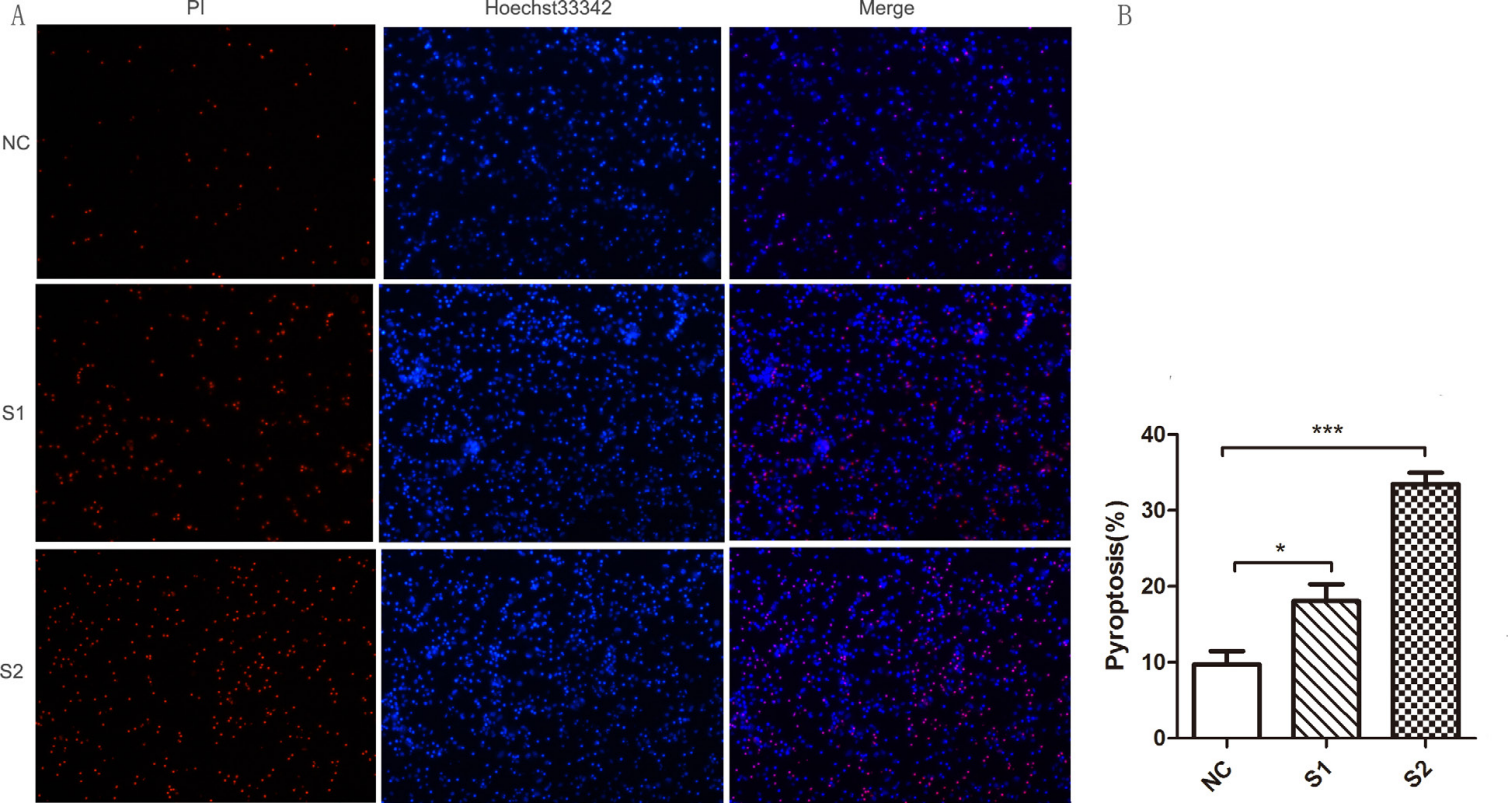
Pyroptosis assay of monocytes infected with ALV-J clinical strains S1 and S2 (**A**) Monocytes isolated from SPF chickens were infected with ALV-J clinical strains S1(1.6 × 10^3^ TCID_50_/0.1mL) and S2 (5.0 × 10^3^ TCID_50_/0.1mL). Images were analyzed as in 5A, above. (**B**) Pyroptosis of five random images containing all cells were calculated. ^*^P < 0.05; ^***^P < 0.001.

**Figure 7:**
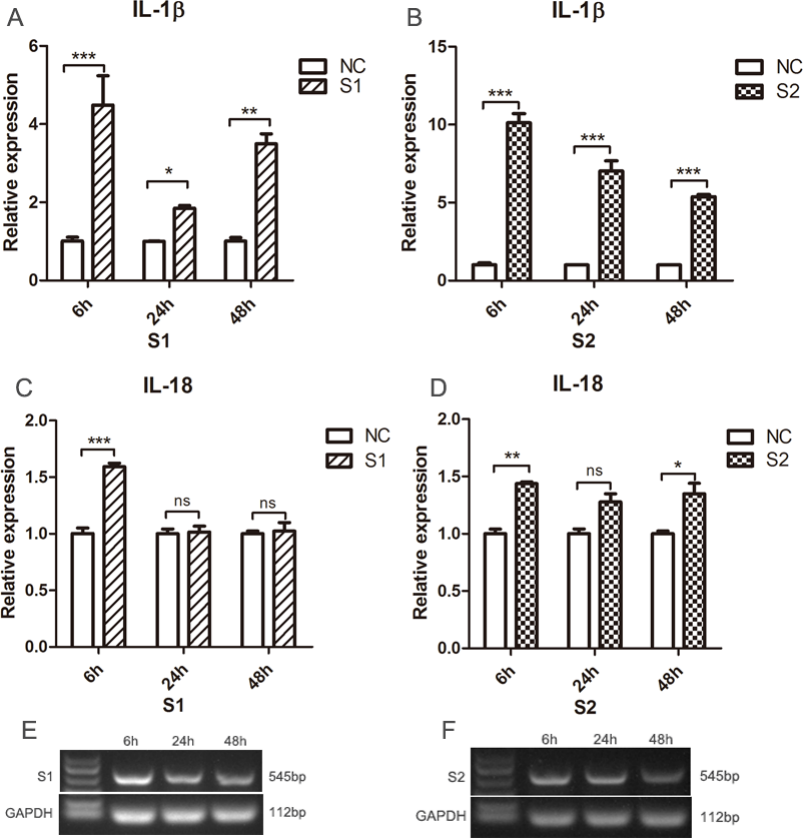
Detection of the pyroptosis-related cytokines expression levels in monocytes infected with ALV-J clinical strains S1 and S2 Monocytes isolated from SPF chicken were infected with ALV-J clinical strains S1(1.6 × 10^3^ TCID_50_/0.1mL) and S2 (5.0 × 10^3^ TCID_50_/0.1mL). The expression of IL-1β (**A**, **B**) and IL-18 (**C**, **D**) were analyzed using qPCR at 6, 24 and 48 hpi. Monocytes at 6, 24 and 48 hpi were collected for RT-PCR using ALV-J specific primers for S1 and S2 (**E**, **F**). NC represented the natural control. ^*^P < 0.05; ^**^P < 0.01; ^***^P < 0.001; ns, not significant.

We also infected SPF chicken monocytes with the ALV-J field strains S1 (1.6 × 10^3^ TCID_50_/0.1 mL) and S2 (5.0 × 10^3^ TCID_50_/0.1 mL) and subjected them to the same monocytes death assay. We found ALV-J field strains S1 and S2 could also induce significant monocyte death (Figure 6A and 6B). Monocytes were successfully infected by ALV-J field strains S1 and S2 from 6 to 48 hpi (Figure 7E and 7F). IL-1β expression was significantly higher in the monocytes infected with S1 or S2 than that in uninfected monocytes from 6 to 48 hpi (Figure 7A and 7B). IL-18 levels in the monocytes infected with field virus S1 were significantly increased at 6 hpi (Figure 7C). IL-18 expression induced by field strain S2 increased from 6 to 48 hpi, but significant differences were found only at 6 and 48 hpi (Figure 7D).

**Figure 8:**
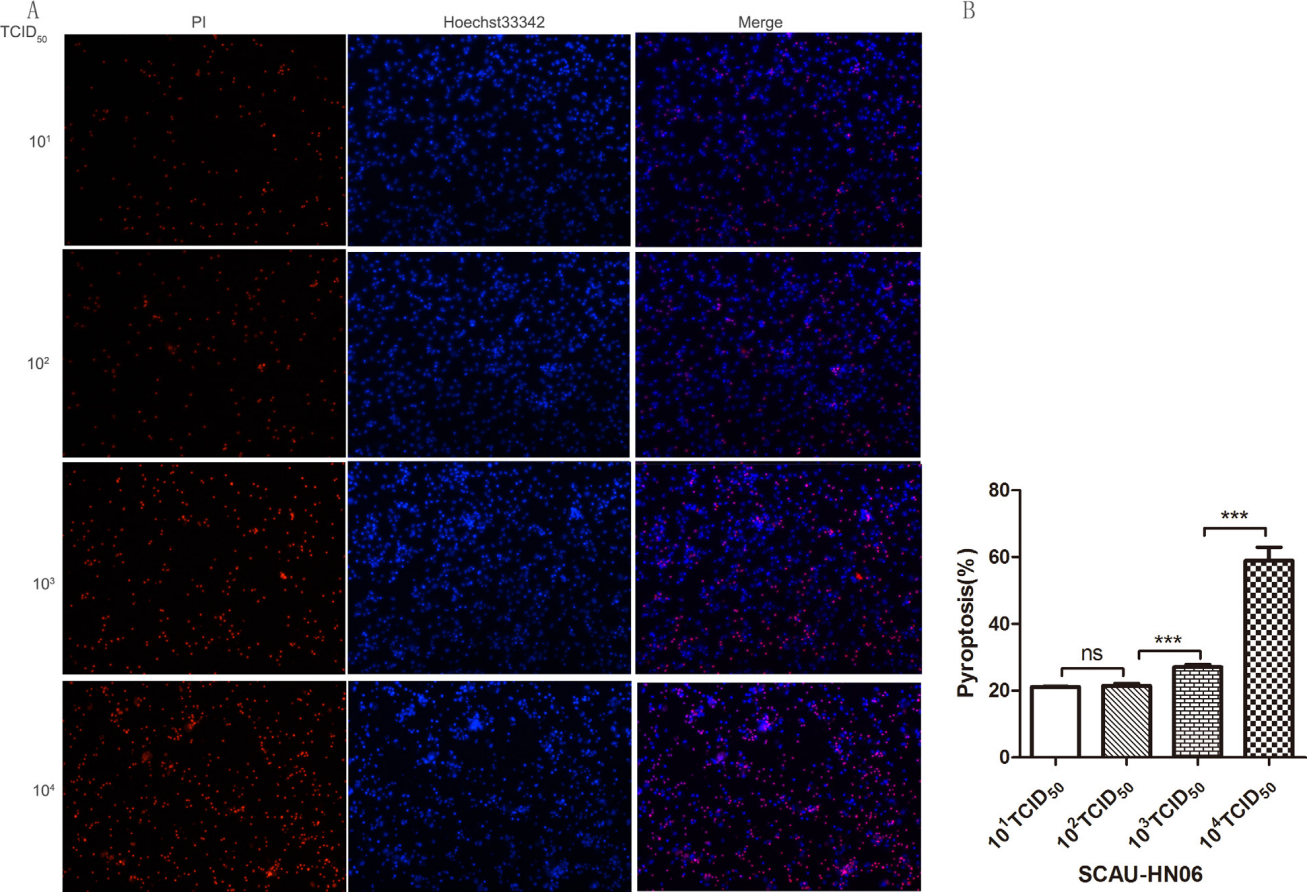
Pyroptosis assay of monocytes infected with different dose of ALV-J strain SCAU-HN06 Monocytes isolated from SPF chickens infected with SCAU-HN06 at different virus titers (10^1^TCID_50_/0.1 mL∼10^4^TCID_50_/0.1 mL). Cell staining and quantification were the same as Figure 5 above.

**Figure 9:**
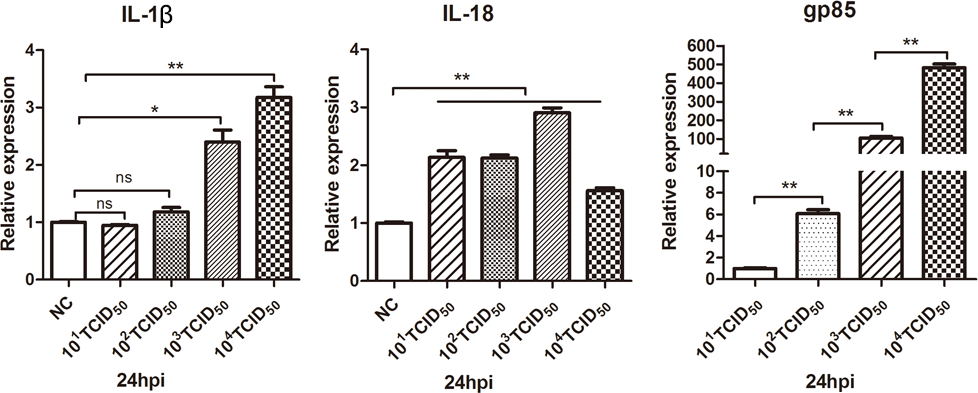
Detection of pyroptosis-related cytokines mRNA levels in monocytes infected with different doses of ALV-J Monocytes isolated from SPF chickens were infected with different doses of ALV-J strain SCAU-HN06. Expression levels of IL-1β, IL-18 and gp85 were analyzed using qPCR at 24 hpi. NC represented the natural control. ^*^P < 0.05; ^**^P < 0.01; ns, not significant.

We further investigated whether ALV-J-induced chicken monocyte death was related to viral titer. We therefore examined the cell death rates and mRNA levels of IL-1β and IL-18 at 24 hpi at different viral titers (10^1^ TCID_50_/0.1 mL to 10^4^TCID_50_/0.1 mL). The death rate of infected monocyte dramatically increased from 10^2^ TCID_50_/0.1 mL to 10^4^ TCID_50_/0.1 mL (Figure 8A and 8B). There was also a positive correlation between the virus titer and mRNA expression levels of IL-1β or viral gp85. It was found that only virus titer reached to 10^3^ and 10^4^ TCID_50_/0.1 mL could induce significant level of IL-1β expression (Figure 9). Interestingly, from titers of 10^1^ to 10^4^ TCID_50_/0.1 mL, there was a significant increase in IL-18 mRNA levels but this was independent of the virus infection level (Figure 9).

Collectively, these results demonstrated that ALV-J infection induced chicken monocyte death in a virus titer-dependent manner associated with up-regulated of IL-1β and IL-18 expression.

### Caspase-1 and caspase-3 activity assay

**Figure 10:**
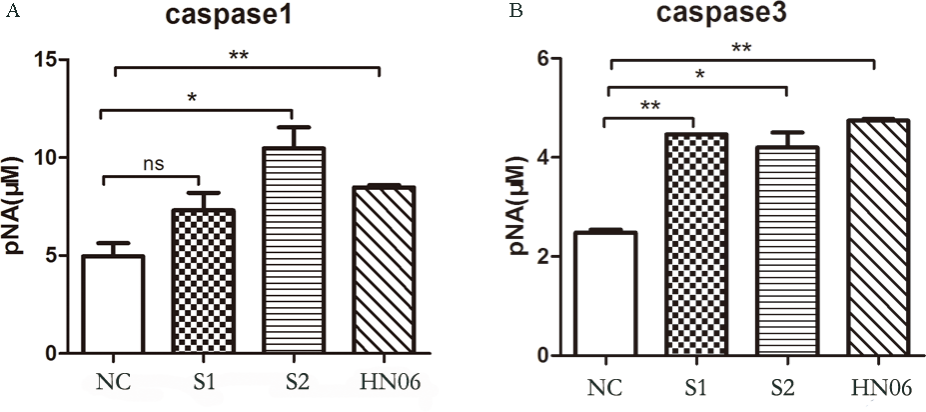
Caspase-1 and caspase-3 activity assay in monocytes infected with ALV-J at 6 hpi Monocytes isolated from SPF chickens were infected with ALV-J strains S1, S2 and SCAU-HN06 for 6 h. NC represents the natural control. Caspase-1 (**A**) and caspase-3 (**B**) activity were measured via a commercial caspase-1 and caspase-3 Activity Assay Kit according to the manufacturer’s instructions. ^*^P < 0.05; ^**^P < 0.01; and ns, not significant.

To confirm whether caspase-1 and caspase-3 were involved in monocyte death induced by ALV-J, caspase-1 and caspase-3 activity were measured at 6 hpi. The ALV-J strain SCAU-HN06 and S2 could significantly increase the level of activated caspase-1 compared with controls (Figure 10A). Field ALV-J strain S1 also could increase the level of activated caspase-1, but did not reach a significant level (Figure 10A). Similarly, ALV-J strains infection including SCAU-HN06, S1 and S2 could significantly enhance caspase-3 activity in infected monocytes. These results demonstrated that ALV-J infection up-regulates caspase-1 and caspse-3 activity in monocytes.

## DISCUSSION

Monocytes are critical effectors and regulators of inflammation and the innate immune response, and they circulate in the bone marrow, spleen and blood [23]. They can also migrate from blood to tissues and differentiate into DCs and macrophages during infection [24]. However, chicken monocyte fate is unknown after ALV infection. In this study, we demonstrated that ALV-J infection induces chicken monocytes death, and the forms of cell death are most likely to be pyroptosis.

This study originated from an interesting phenomenon that most monocytes isolated from clinical chicken infected with ALV-J could not differentiate into macrophages due to cell death. Eukaryotic cell death can be caused by a wide variety of pathogenic microorganisms [25]. Cell death programs include apoptosis, autophagy, necrosis, oncosis and pyroptosis [16, 25]. Pyroptosis could be triggered by various pathogenic microorganism infections accompanied by inherently inflammatory [16]. Given that monocytes are key players during inflammation and pathogen challenge, we speculated that monocyte death caused by ALV-J was associated with inflammation [26]. Therefore, pyroptosis in the monocytes isolated from SPF chickens was analyzed after ALV-J infection.

We demonstrated that caspase-1 was activated at 6 hpi. Besides, the IL-1β expression level at 6, 24 and 48 hpi, and IL-18 expression level at 6 and 24 hpi were all significantly increased during SCAU-HN06 infection. The inflammatory cytokines IL-1β and IL-18 undergo secretion with caspase-1-dependent activation during pyroptosis [16]. Due to a lack of commercial antibodies for chickens, we could not measure caspase-1 activation and IL-1β production by Western blot. Caspase-1 activation results in pore formation in the plasma membrane, and the cell becomes permeable to small molecular weight, membrane-impermeable dyes such as PI [27]. In contrast, apoptotic cells remain intact and are not stain with PI in the early stage of apoptosis [27]. PI staining has been used for pyroptosis assay [28]. In this study, cell death rate in ALV-J infected monocytes was significantly higher than the rate in control monocytes analyzed with PI staining. Despite the lack of critical experiments such as cleavage of gasdermins and activation of caspase-1 in protein level, we can still speculate that the chicken monocyte death induced by ALV-J infection would most likely to be pyroptosis based on current evidence.

NLRP3 expression was not statistically altered in this study. Pyroptosis-related inflammasomes such as NLRP3, NLRC4 and AIM2 recruit and activate caspase-1 in response to microbial stimuli [12, 14]. NLRP3 has been most extensively studied and many different strains of virus including ALV-J can activate the NLRP3 inflammasome [29, 30]. Our results showed that NLRP3 was not involved in the process of monocyte death. However, chickens lack all DNA-responsive PYHIN proteins including AIM2 [31, 32], which makes it impossible to be involved in monocyte pyroptosis induced by ALV-J. In addition, GSDMD is not identified in chickens, and chicken just possess GSDMA (NCBI Reference Sequence: NM_001031361.1), DFNA5 (NM_001006361.1) and DFNB59 (XM_426573.5). Identification of GSDMD as a pyroptotic substrate of inflammatory caspases is a revolutionary transition in the understanding of pyroptosis. However, the function of other gasdermins and their mechanisms of activation are still unknown [19]. Therefore, future studies to explore which innate immune sensor and pyroptotic substrate are involved in pyroptosis in chickens are worthwhile.

Besides, many viruses, including Hepatitis C, influenza and Dengue have been verified to simultaneously cause pyroptosis and apoptosis in infected or bystander cells [33–35]. PI can stain late apoptotic cells and caspase-3 will be activated in the apoptotic cell. Interestingly, these phenomena appeared in our results. Therefore, we speculate that ALV-J infection also could simultaneously induce pyroptosis and apoptosis in chicken monocytes, which needs to be further verified in future.

One interesting phenomenon in this study is that almost all monocytes isolated from clinical chickens infected with ALV-J had died in subsequent culture. We suspect that this phenomenon was due to high ALV-J titers in these animals. However, this did not correlate with the relatively modest levels of viremia that we measured. Furthermore, the monocyte death induced by ALV-J clinical strains was less than that induced with the control ALV-J strain SCAU-HN06. Our results demonstrated that chicken monocyte death induced by ALV-J infection is viral titer dependent. However, ALV-J clinical strain S1 could not significantly activate caspase-1. Field strains isolated from animals often possess lots of variants and exist as a complex mixture of different, but closely related genomes named quasispecies [36]. However, infectious clone of ALV-J (laboratory strains) has a single origin that was constructed from a full-length copy of the proviral genome from field strain [36]. The quasispecies phenomena may be primarily caused by viral variations and affect the clinical manifestations of patients and the antiviral therapeutic response [37, 38]. These may be the reasons for the distinction of the three ALV-J strains presented in this study.

ALV-J infections cause enormous economic losses in the poultry industry all over the world [39]. However, there are still no effective vaccines or drugs to protect against ALV-J infections in chickens. ALV-J can cause tumors as well as immunosuppression and the mechanisms of immunosuppression are still not fully understood. Monocyte death induced by ALV-J infection could directly cause a decrease in monocytes and further lead to decreases in macrophages and DCs. All three of these cell types are extremely important for resistance to viral invasion. Reducing their numbers will lead to a decline in immune function. This could be an important cause of immunosuppression in chickens infected with ALV-J. In addition, pyroptosis could play a role as a protective host response to infectious diseases by removing intracellular pathogens and releasing pro-inflammatory cytokines [16, 30]. This mode may be a useful avenue to approach defense strategies against ALV-J infection. However, virus-related pyroptosis researches are still rare and the mechanism of pyroptosis in chicken cells is also still unknown. Our present work is just a beginning, and the mechanisms of pyroptosis in chicken cells and avian virus-related pyroptosis require more investigations.

In summary, we demonstrated that ALV-J infection can induce chicken monocytes death, and the form of cells death may be a mixture including pyroptosis and apoptosis.

## MATERIALS AND METHODS

### Ethics statement

Chicken blood samples were collected in this study. All animal experiments obtained approval and guidance from South China Agriculture University Institutional Animal Care and Use Committee.

### Virus

ALV-J laboratory strain SCAU-HN06 is an infectious clone of ALV-J that was constructed from a full-length copy of the proviral genome from field strain SCAU-HN06 isolated by our laboratory from commercial Roman layers with spontaneous haemangiomas [21, 40].

### Sample collection

Four Chinese yellow chickens were collected from a farm in Guangdong Province, China. Two of them (designated S1, S2) were suspected infection of ALV-J based on symptoms of obvious hemangiomas on the surface of skin. The remaining two normal chickens were designated N1 and N2. In addition, 4- to 8- week old specific-pathogen-free (SPF) White Leghorn chickens (half males and half females) were purchased from Guangdong Dahuanong Animal Health Products (Guangzhou, China) and housed in isolator cages.

Blood samples were collected aseptically with anticoagulant and centrifuged at 1, 200 rpm for 15 min to separate upper plasma samples and cell pellets. The plasma samples were stored at −80°C for ALV detection. Cell pellets were used to isolate the peripheral blood mononuclear cells (PBMC) by lymphocyte separation medium (Solarbio, Beijing, China).

### Enzyme-linked immunosorbent assay (ELISA) for ALV

The plasma samples were inoculated into DF1 (American Type Culture Collection, Manassas, VA, USA) cell cultures in 24-well plates (Corning, USA). The cells were maintained at 37°C with 5% CO_2_ for 5–7 d, and then frozen and thawed three times. Supernatants were harvested after centrifugation at 1,000 rpm for 5 min. The supernatants were tested for ALV group-specific antigen (p27) using the Avian Leukosis Virus Antigen Test Kit (Idexx Bioresearch, USA) according to the manufacturer’s instructions. The results were expressed as s/p ratios where s/p = (Sample Mean –Kit Negative Control Mean) /(Kit Positive Control Mean –Kit Negative Control Mean). Viremia of the four Chinese yellow chickens was monitored once a week for three weeks.

### Culture conditions of clinical yellow chicken monocytes

According to previously described methods [4, 41], the isolated PBMC of clinical chickens were incubated for 6 h at 37°C with 5% CO_2_. Then the supernatants were removed and adherent cells were washed twice with warm PBS to remove thrombocytes, non-adherent lymphocytes and other semi-adherent cells. The remaining adherent cells were primarily chicken monocytes. Fresh RPMI-1640 medium (15% chicken serum, 100 U/mL penicillin and 100 mg/mL streptomycin) was added to the monocytes, and they were cultured for 6 days to allow macrophage differentiation. The culture medium was changed every 2 d in order to ensure stable and consistent conditions.

### Infection of SPF chicken monocytes with ALV-J

PBMC isolated from SPF chickens was infected with a dose of 0.1 mL (10^4.5^ TCID_50_/0.1 mL) of laboratory ALV-J strain SCAU-HN06. After 6 h of incubation, supernatants were removed and monocytes were then cultured for 6 d to maturity for macrophage differentiation. The culture medium was changed every 2 d in order to ensure stable and consistent conditions. SPF chicken monocytes were infected with field ALV-J strains isolated from S1 and S2 using the same method.

### Western blot analysis

Western blotting was performed following our previously described method with ALV-J envelope protein specific mouse anti-monoclonal antibody JE9 (kindly provided by Dr. Aijian Qin, Yangzhou University, Yangzhou, China) and rabbit anti-β-actin antibody [22]. IRDye 700DX-conjugated anti-rabbit IgG and IRDye 800-conjugated anti-mouse IgG (Rockland Immunochemicals, Limerick, PA, USA) were used as the secondary antibody. Membranes were visualized and analyzed with an Odyssey infrared imaging system (LI-COR Biosciences, Lincoln, NE, USA).

### Pyroptosis assay

Cell pyroptosis was measured as described previously [28]. Briefly, monocytes were isolated from SPF chickens and infected with ALV-J. The cells were stained with PI (2 µg/ml) and Hoechst 33342 (5 µg/ml) (Beyotime Biotechnology, Shanghai, China) at 24 hours post infection (hpi). Dead cells (PI permeable, red) were observed under a fluorescence microscope (Nikon, Japan) using NIS-Elements BR analysis software (Nikon, Japan).

### mRNA quantification

Total RNA was extracted from infected monocytes at 6, 24 and 48 hpi using an RNAfast200 kit (Fastagen, Shanghai, China). cDNA synthesis was carried out using a PrimeScript RT Reagent Kit (Takara, Japan) according to the manufacturer’s protocol. qPCR primers for IL-1β , IL-18 and NLRP3 have been reported previously [29]. The GAPDH gene was used as an internal control. qPCR was performed on a Bio-Rad CFX96 Real-Time Detection System using iTaqTM Universal SYBRGreen Supermix Kit reagents (Bio-Rad, CA, USA). Data analyses were performed using the 2^−ΔΔCt^ method [42]. RT-PCR was employed to detect ALV-J infection using specific primers [43].

### Caspase-1 and caspase-3 activity assay

Caspase-1 activity was measured using a commercial Caspase-1 Activity Assay Kit according to the manufacturer’s instructions (Beyotime, China). This assay is based on the ability of caspase-1 to change acetyl-Tyr-Val-Ala-Asp *p*-nitroaniline (Ac-YVAD-pNA) into the yellow formazan product *p*-nitroaniline (pNA). Total cytosolic protein was extracted from ALV-J infected monocytes at 6 hpi and incubated in a 96-well microtiter plate with 20 nMol Ac-YVAD-pNA for 2 h at 37°C. Absorbance values were measured at 405 nm. Uninfected monocytes were used as a control. Similarly, caspase-3 activity was measured using the Caspase-3 Activity Assay Kit according to the manufacturer’s instructions (Beyotime, China).

### Statistical analyses

Statistical comparisons were performed using GraphPad Prism 5 (GraphPad Software Inc., USA). Results are presented as means ± SEM, and statistical significance was assessed at *P value*s of < 0.05, 0.01, or 0.001.

## SUPPLEMENTARY MATERIALS FIGURE


