# Supplementary material for: ALV-J infection induces chicken monocyte death accompanied with the production of IL-1β and IL-18
Source: Oncotarget. 2017 Oct 13;8(59):99889–900. doi: 10.18632/oncotarget.21906 (PMC5725138; doi:10.18632/oncotarget.21906)
Supplement: Supplementary file 1 [file oncotarget-08-99889-s001.pdf]

ALV-J infection induces chicken monocyte death accompanied with the production of IL-1β and IL-18

SUPPLEMENTARY MATERIALS

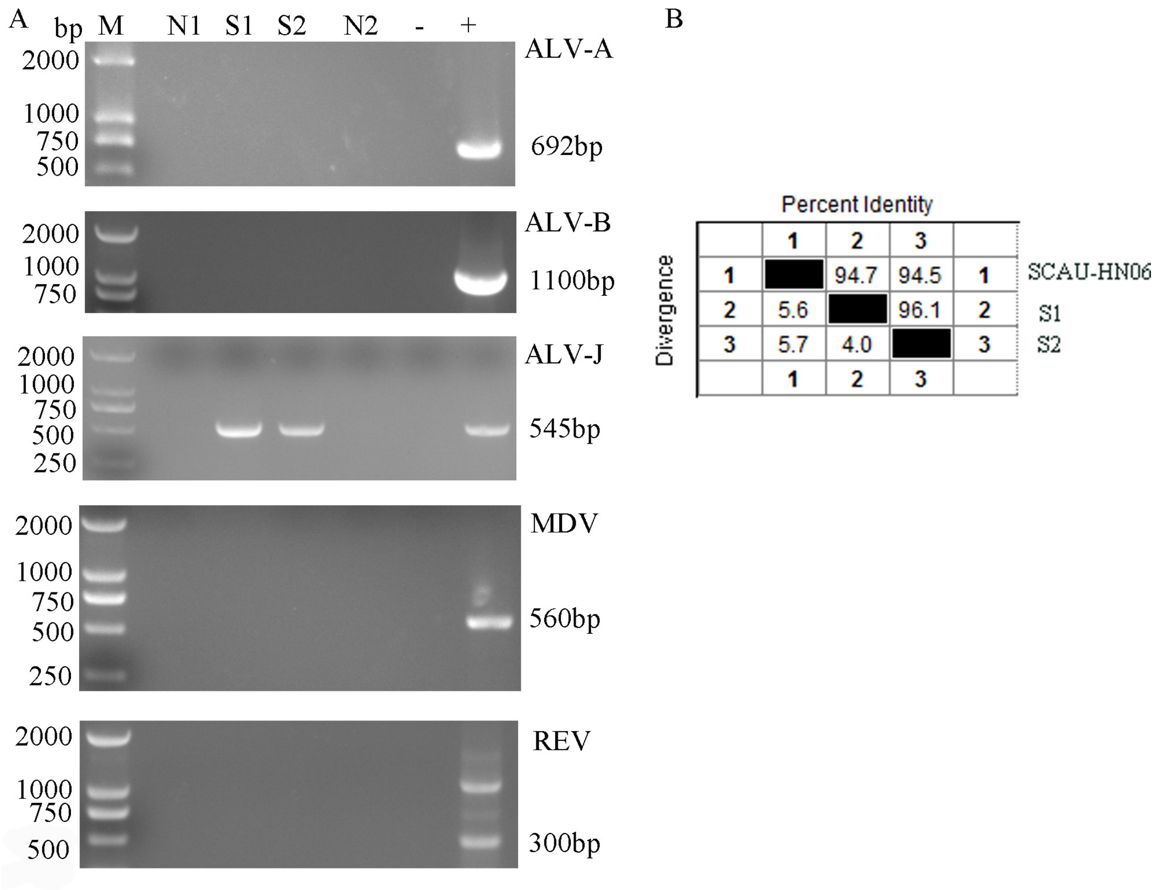

Supplementary Figure 1: ALV infections detected by specific PCR and analysis of env sequence of ALV strain S1 and S2. DNA was extracted from the DF1 cells incubated with the plasma of N1, N2, S1 and S2 samples using a commercial kit (Omega, USA). (**A**) PCR results using primers specific for MDV, REV, ALV-J, ALV-A and ALV-B. S1 and S2 samples produced ALV-J specific 545 bp fragment. bp = base pairs, M = Marker. (**B**) Sequence alignments were compared between the env genes of S1, S2 and SCAU-HN06. The nucleotide sequences were aligned using the MegAlign function in the sequence analysis software Lasergene (version 7.10) (DNASTAR, Madison, WI, USA).
